# Endoscopic Approach to Postoperative Maxillary Cysts Ensuring Infraorbital Nerve Safety With Direct Approach to the Anterior and Lateral Part of the Maxillary Sinus With an Endoscope (DALMA): A Case Report

**DOI:** 10.7759/cureus.69490

**Published:** 2024-09-15

**Authors:** Kosuke Takabayashi, Kosuke Akiyama

**Affiliations:** 1 Otorhinolaryngology, Japanese Red Cross Asahikawa Hospital, Asahikawa, JPN; 2 Otorhinolaryngology, School of Medicine, Sapporo Medical University, Sapporo, JPN; 3 Otolaryngology and Head and Neck Surgery, Kagawa University, Takamatsu, JPN

**Keywords:** caldwell-luc surgery, endoscopic sinus surgery, nasolacrimal duct, pedicled mucosal flap, piriform aperture

## Abstract

Postoperative maxillary cysts (POMCs) present significant challenges, especially when located laterally or when the infraorbital nerve's course is unclear on imaging. Traditional endoscopic approaches are often limited by a high risk of recurrence and potential nerve injury. Here, we report a case of a 72-year-old woman with a right maxillary cyst, which caused pain and swelling. Imaging revealed a cyst with infraorbital wall defects and uncertain nerve positioning. Using the direct approach to the anterior and lateral maxillary sinus with an endoscope (DALMA), we successfully identified and preserved the infraorbital nerve while opening the cyst wall. A mucosal flap from the nasolacrimal duct was utilized to cover the cyst opening, secured by suturing with the medial cyst wall to prevent recurrence. Postoperative recovery was uneventful, with significant relief of symptoms. Eight-month follow-up imaging confirmed an open cyst without recurrence or nerve damage.

This case demonstrates the efficacy of the DALMA technique in managing complex POMCs, offering a safe and effective method for cases with challenging anatomical features. Using a nasolacrimal duct mucosal flap provided the necessary coverage and stability to prevent recurrence. This approach significantly reduces the risk of infraorbital nerve injury and recurrence, making it a valuable advancement in endoscopic sinus surgery.

## Introduction

Surgical treatment of postoperative maxillary cysts (POMCs) can effectively prevent recurrence by placing a mucosal flap over the cyst opening [1-3]. Additionally, the development of the prelacrimal approach has expanded the indications for endoscopic nasal sinus surgery in managing POMCs [4,5]. However, the risk of recurrence remains high for laterally located cysts [1,6]. Caution should be exercised if the infraorbital nerve runs medially to the cyst or is not discernible on imaging, as the risk of nerve damage is significant [7].

A recently developed technique allows for more lateral surgical access into the maxillary sinus compared to the prelacrimal approach, enabling endoscopic endonasal surgery for laterally located cysts [6]. This technique also exposes the anterior wall of the maxillary sinus, providing clear visualization of the infraorbital nerve during surgery [6].

No previous reports have provided a detailed step-by-step account of surgical findings in cases involving laterally located cysts with an unclear infraorbital nerve course on preoperative imaging. In this case, the direct approach to the anterior and lateral part of the maxillary sinus with an endoscope (DALMA) technique [6] was performed to clearly identify the course of the infraorbital nerve, allowing the cyst to be opened without causing neural injury. Furthermore, since the nasal cavity mucosal flap did not reach the cyst opening, the nasolacrimal duct mucosa was used as a mucosal flap, which was sutured and secured to the cyst mucosa. This case report provides a detailed account of the treatment.

## Case presentation

**Figure 1:**
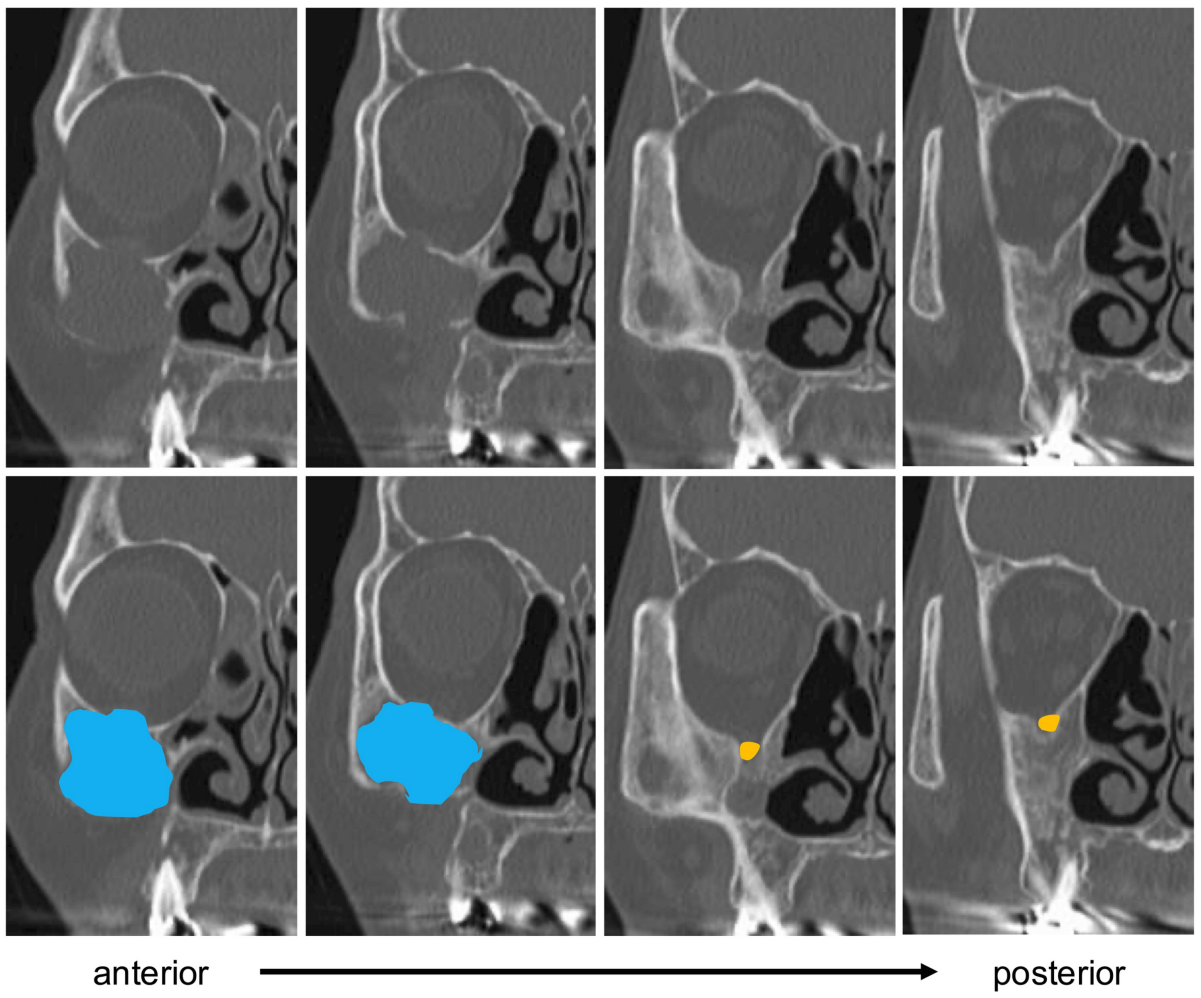
Preoperative computed tomography (CT) images The right side is anterior, and the left side is posterior as one moves toward the left. The upper panel shows the plain CT image, while the lower panel highlights the cyst in blue and the nerve in orange. It was not possible to identify the exact location of the infraorbital nerve.

**Figure 2:**
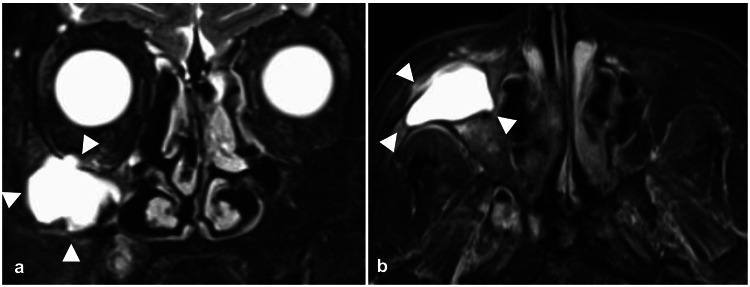
Preoperative magnetic resonance imaging (MRI) T2-weighted images are shown. A structure with a clear border from the surrounding area and a nearly homogeneous internal water signal was identified in the right maxilla and diagnosed as a postoperative maxillary cyst (POMC). White arrowheads indicate the POMC. (a) shows a coronal slice and (b) shows an axial slice.

A 72-year-old woman presented with pain and swelling extending from the right cheek to the gingiva, which has persisted for three months. She had undergone Caldwell-Luc surgery 50 years earlier. Computed tomography (CT) images revealed a lobulated spherical cystic structure in the right maxilla, with partial infraorbital wall defects and protrusion into the orbit from the same area. The precise location of the infraorbital nerve could not be identified (Figure 1). Magnetic resonance imaging (MRI) showed a cystic structure in the right maxilla with a clear border from the surrounding area and a largely homogeneous fluid signal inside, confirming the diagnosis of a POMC (Figure 2). 

**Figure 3:**
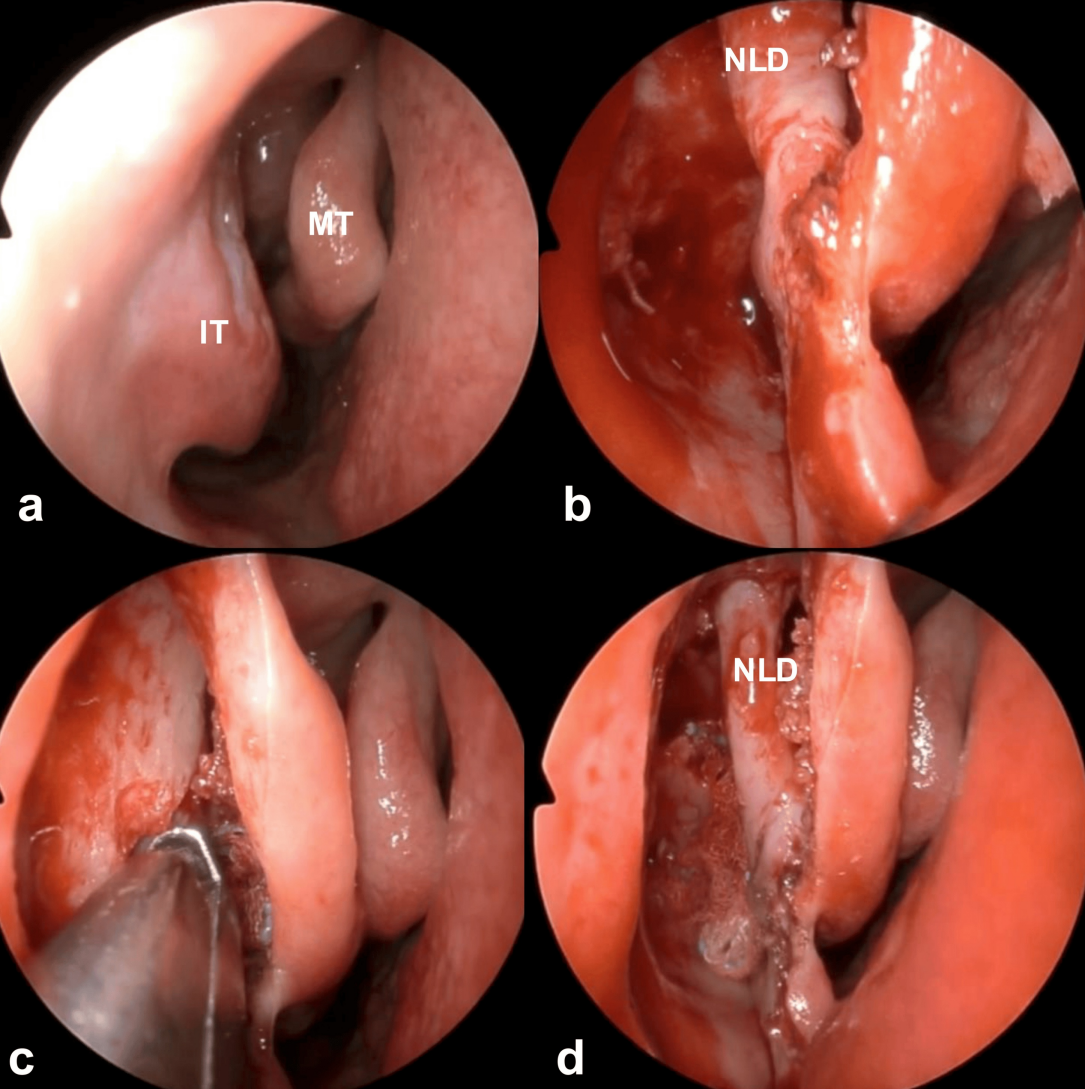
Intraoperative endoscopic findings 1 Portions of the maxillary and lacrimal bones were excised using a prelacrimal approach through a mucosal incision along the piriform aperture (a-c), and the mucosa of the lateral wall of the nasal cavity and the nasolacrimal duct were shifted medially (d). IT: inferior turbinate; MT: middle turbinate; NLD: nasolacrimal duct

**Figure 4:**
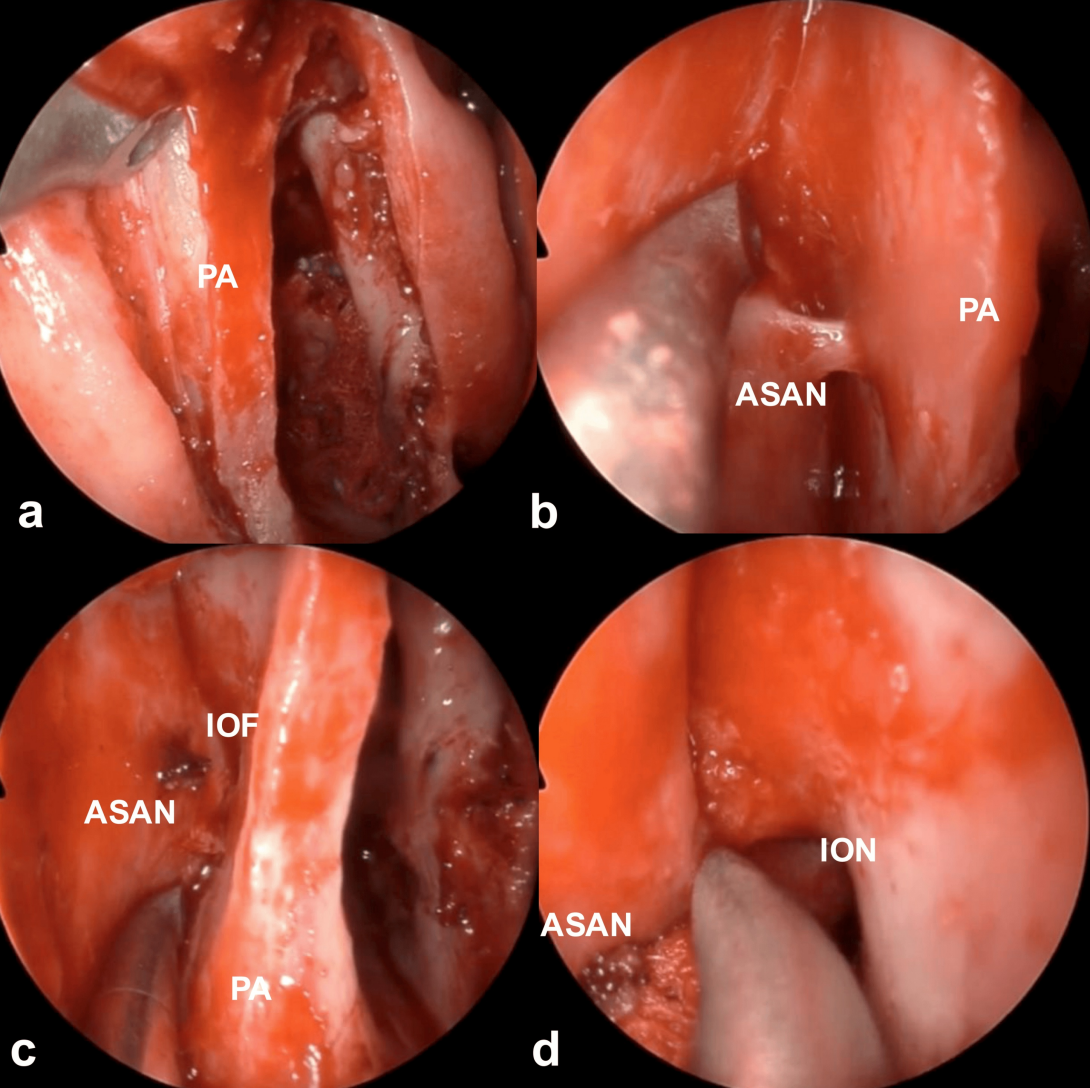
Intraoperative endoscopic findings 2 Elevation of the skin from the anterior wall of the maxillary sinus at the mucosal incision site near the piriform aperture identified the infraorbital foramen lateral to and above the anterior superior alveolar nerve (a-c). The cyst wall was resected while clearly identifying and preserving the infraorbital nerve (d). ASAN: anterior superior alveolar nerve; IOF: infraorbital foramen; ION: infraorbital nerve; PA: piriform aperture

**Figure 5:**
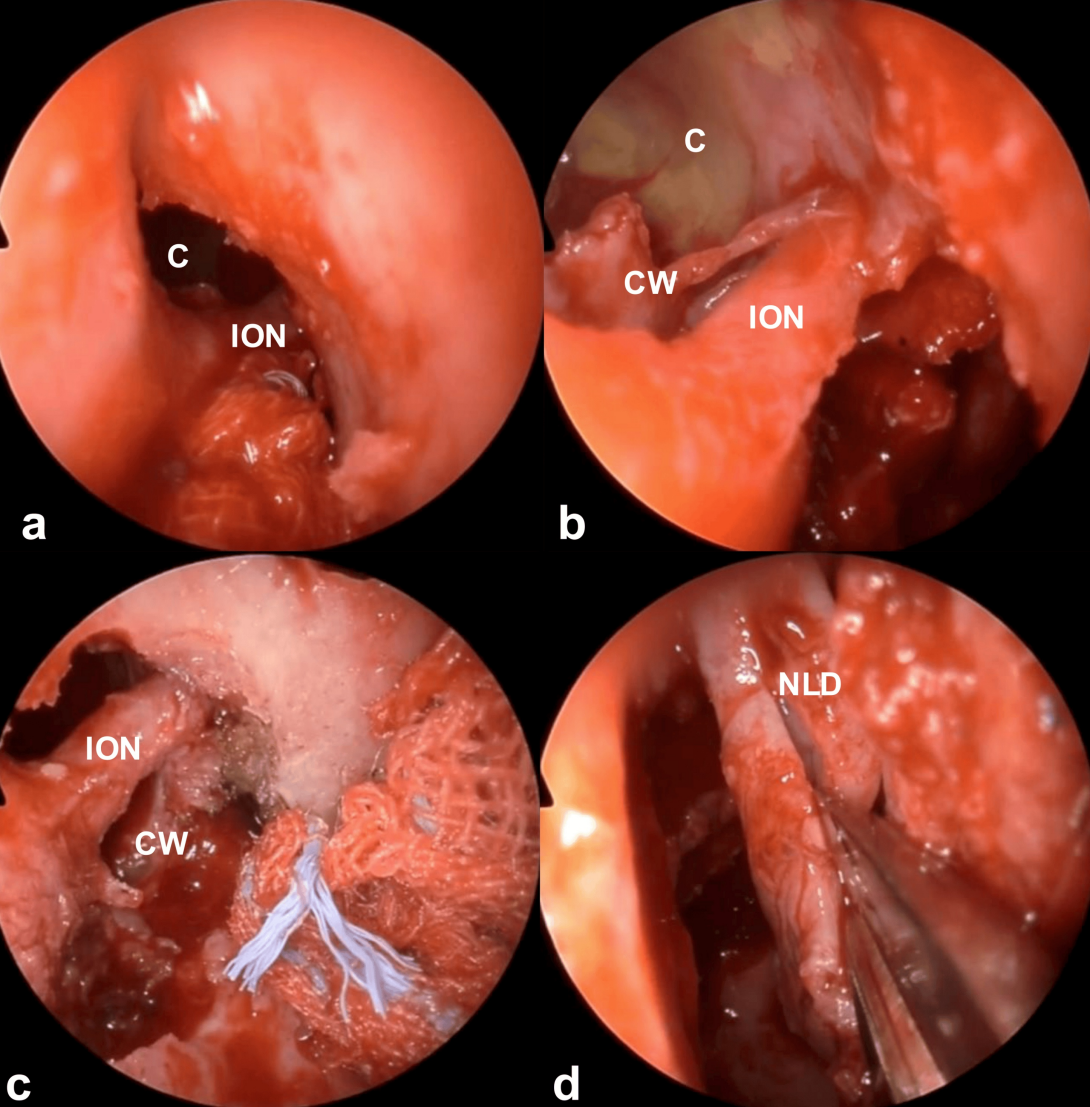
Intraoperative endoscopic findings 3 Manipulation was continued with the identification and preservation of the infraorbital nerve. The medial cyst wall was inverted nasally and sutured to a pedicled mucosal flap to prevent recurrence (a-c). The nasolacrimal duct was longitudinally incised to be utilized as a mucosal flap (d). C: cyst; CW: cyst wall; ION: infraorbital nerve; NLD: nasolacrimal duct

**Figure 6:**
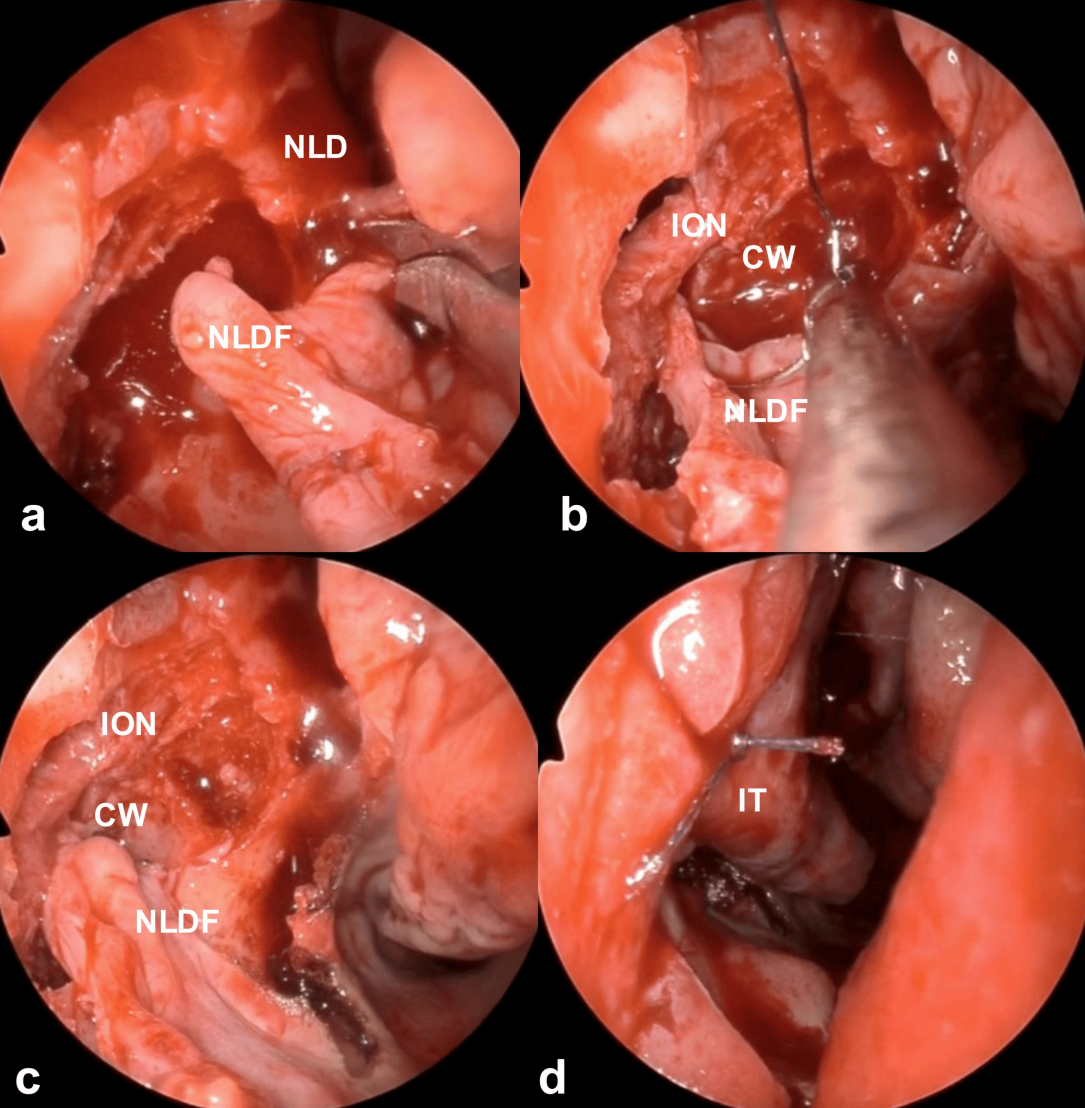
Intraoperative endoscopic findings 4 The mucosa from the lateral wall of the nasal cavity to the lateral wall of the nasolacrimal duct, based on the nasal floor, was harvested as a pedicled mucosal flap to prevent recurrence of the cyst (a). The flap from the lateral wall of the nasal cavity to the lateral wall of the nasolacrimal duct and the medial cyst wall were sutured (b, c). Finally, the operation was concluded by suturing the mucosal incision in the piriform aperture (d). CW: cyst wall; ION: infraorbital nerve; IT: inferior turbinate; NLD: nasolacrimal duct; NLDF: nasolacrimal duct flap

Endoscopic endonasal surgery was performed to relieve the patient's primary symptoms of pain and swelling. Portions of the maxillary and lacrimal bones were excised using a prelacrimal approach through a mucous membrane incision along the piriform aperture. The mucosa of the lateral wall of the nasal cavity and the nasolacrimal duct were shifted medially (Figures 3a-3d). Elevation of the skin from the anterior wall of the maxillary sinus at the mucosal incision site near the piriform aperture exposed the infraorbital foramen, located lateral to and above the anterior superior alveolar nerve (Figures 4a-4c). The cyst wall was resected while clearly identifying and preserving the infraorbital nerve (Figures 4d, 5a). The medial cyst wall was inverted nasally to prevent recurrence and sutured to a pedicled mucosal flap (Figures 5b, 5c). A pedicled mucosal flap was harvested from the lateral wall of the nasal cavity to the lateral wall of the nasolacrimal duct, based on the nasal floor, to prevent cyst recurrence (Figures 5d, 6a). This flap, along with the medial cyst wall, was sutured (Figures 6b, 6c). Finally, the procedure was concluded by suturing the mucosal incision at the piriform aperture (Figure 6d, Video 1).

**Video 1:** Surgical procedure Portions of the maxillary and lacrimal bones were excised using a prelacrimal approach through a mucosal incision along the piriform aperture, and the mucosa of the lateral wall of the nasal cavity and the nasolacrimal duct were shifted medially. Elevation of the skin from the anterior wall of the maxillary sinus at the mucosal incision site near the piriform aperture identified the infraorbital foramen lateral to and above the anterior superior alveolar nerve. The cyst wall was resected while clearly identifying and preserving the infraorbital nerve. Manipulation was continued with the identification and preservation of the infraorbital nerve. The medial cyst wall was inverted nasally and sutured to a pedicled mucosal flap to prevent recurrence. The mucosa from the lateral wall of the nasal cavity to the lateral wall of the nasolacrimal duct, based on the nasal floor, was harvested as a pedicled mucosal flap to prevent recurrence of the cyst. The flap from the lateral wall of the nasal cavity to the lateral wall of the nasolacrimal duct and the medial cyst wall were sutured. Finally, the operation was concluded by suturing the mucosal incision in the piriform aperture.

**Figure 7:**
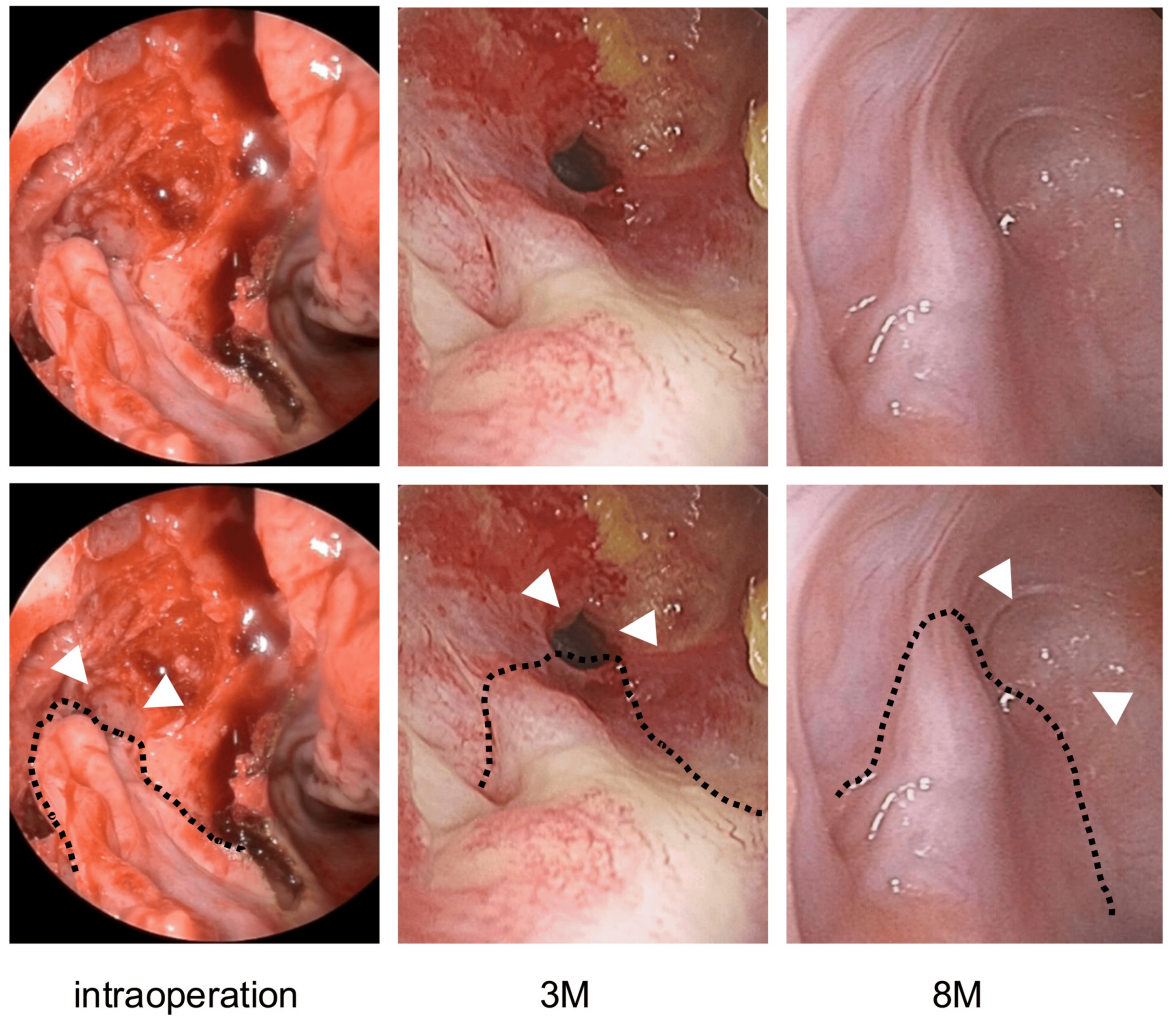
Endoscopic findings of the cyst opening In the upper panel, plain endoscopic images are shown, while in the lower panel, the pedicle flap is dotted, and the cyst opening is highlighted with a white arrowhead. The lower section of the lower panel indicates the postoperative time. The pedicled mucosal flap was engrafted successfully, contributing to the cyst remaining open. M: months

**Figure 8:**
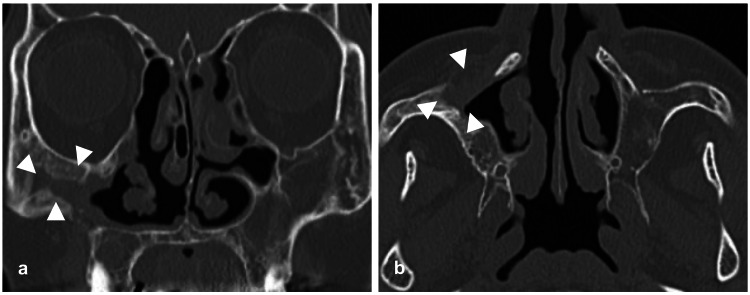
Postoperative CT images The cyst remained open and spontaneously contracted. White arrowheads indicate the cyst. (a) shows a coronal slice and (b) shows an axial slice.

**Figure 9:**
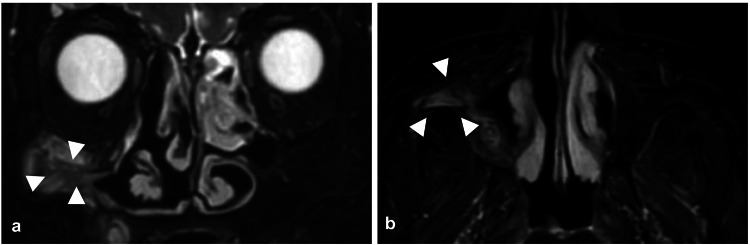
Postoperative MRI T2-weighted images are shown. The high-intensity area indicating postoperative maxillary cyst (POMC), observed preoperatively, disappeared. White arrowheads indicate the cyst. (a) shows a coronal slice and (b) shows an axial slice.

Postoperatively, buccal pain and swelling improved, and the pedicled mucosal flap engrafted successfully, contributing to the cyst remaining open (Figure 7). Eight-month follow-up imaging via CT and MRI images confirmed that the cyst remained open and spontaneously contracted (Figures 8, 9). No findings of neuropathy in the infraorbital nerve area were observed.

## Discussion

Endoscopic surgery for POMCs presents challenges, particularly when the cyst is located laterally [1,6] or when the infraorbital nerve’s course is unknown [7]. This case report shows that these difficulties can be addressed effectively by using the DALMA technique. Furthermore, a long mucosal flap is required to cover the open part of the laterally located cyst. This was achieved by using the nasolacrimal duct mucosa as a flap, which provided the necessary length for coverage.

The development of the prelacrimal approach has expanded the scope of endoscopic sinus surgery by allowing manipulation of maxillary sinus lesions located outside the nasolacrimal duct while preserving the inferior turbinate and nasolacrimal duct [4,5]. Additionally, the recent development of the DALMA technique enables precise surgical access to the anterior and lateral walls of the maxillary sinus using a transnasal endoscope [6]. This has made it possible to perform transnasal endoscopic surgery for POMCs, which until now have been difficult to open transnasally. In this case, we successfully treated a POMC located on the anterior wall of the maxillary sinus lateral to the infraorbital foramen, an area typically considered difficult to access transnasally. DALMA provided comprehensive endoscopic visualization of the lesion in the maxillary sinus and facilitated surgical manipulation.

The infraorbital nerve normally runs along the infraorbital wall; however, in surgery for POMCs, its course may be displaced, increasing the risk of injury. Confirming the infraorbital nerve’s course as accurately as possible using intraoperative navigation systems and preoperative CT scans is crucial [7-9]. However, the risk of injury remains high if the infraorbital nerve’s location is unclear on imaging or if it runs medially to the cyst [7]. Performing DALMA exposes the anterior wall of the maxillary sinus via a transnasal endoscope, allowing for precise identification of the infraorbital foramen, which enables safe surgical manipulation with clear visualization of the infraorbital nerve [6]. In maxillary tumor surgery, transposition of the infraorbital nerve by drilling down the maxillary bone was reported as an effective method to protect the nerve [10]. DALMA enabled endoscopic and minimally invasive resection of the bone around the infraorbital nerve, providing mobility to the nerve and reducing the risk of damage. In this case, the patient experienced no sequelae affecting the infraorbital nerve.

Mucosal flaps covering the edges of the open window site of a POMC can prevent re-occlusion [1-3]. However, when the cyst is lateral, the mucosa of the lateral nasal wall may not be long enough to cover the cyst wall. In this case, the nasolacrimal duct was incised longitudinally, and the mucosa of the lateral half was harvested as a mucosal flap. This was then connected to the mucosal flap of the lateral wall of the nasal cavity, creating a longer flap than what could be obtained from the lateral wall alone. Since the cyst was positioned above the nasal floor, there was concern that the mucosal flap covering the cyst wall could be displaced by gravity. To prevent this, the mucosal flap was stabilized and secured by suturing the opened cyst wall to the nasolacrimal duct mucosal flap. DALMA allows the needle holder to reach the outside of the maxillary sinus, making it possible to suture the mucosa, which was previously difficult.

Using DALMA facilitated the safe and effective treatment of a POMC located laterally with an infraorbital nerve course, which had been difficult to treat in the past.

## Conclusions

POMCs, which were traditionally challenging to treat with transnasal endoscopy, can now be treated successfully using the DALMA technique. Even when the infraorbital nerve is poorly visualized on imaging, the risk of nerve injury is reduced by confirming the nerve's position through clear visualization of the infraorbital foramen, allowing safe surgical manipulation. A long mucosal flap can be effectively harvested by combining tissue from the nasolacrimal duct and the lateral nasal wall, ensuring adequate coverage of lateral cysts. Additionally, securing the flap with sutures to the cyst wall improves stability in the open area. We believe that this detailed report will contribute to advancements in the treatment of refractory POMCs.
